# Case Report: Repair of an Iatrogenic Right Carotid-Internal Jugular Vein Fistula

**DOI:** 10.7759/cureus.87199

**Published:** 2025-07-02

**Authors:** Sunny H Vansdadia, Auggie Herber, Medhavi Meka

**Affiliations:** 1 Neurological Surgery, University of Arizona College of Medicine-Phoenix, Phoenix, USA; 2 General Surgery, Banner University Medical Center-Phoenix, Phoenix, USA; 3 Vascular Surgery, Carl T. Hayden Medical Center, Phoenix, USA

**Keywords:** arteriovenous fistula, carotid-jugular fistula, central line catheterization, rare complication, vascular anomaly

## Abstract

A carotid-jugular fistula (CJF) is a direct communication between the carotid artery and jugular vein, which has been described as a rare complication of trauma, central line placement, or surgical intervention. We report a rare case of a patient with a right CJF presumed to be the result of previous central line catheterization. This case report characterizes the vascular pathology and describes the subsequent surgical intervention. The goal of this case report is to provide clinicians with a framework for treating this rare pathology and to contribute to the literature on this devastating potential complication.

## Introduction

Carotid-jugular fistulas (CJF) are extremely rare, and only a limited number of cases have been reported in the literature. Arteriovenous fistulas (AVFs) can be either congenital or acquired and frequently occur as a result of traumatic or iatrogenic causes. CJFs result from direct communication between the carotid artery and the jugular vein. Although rare, they have been reported as complications following penetrating neck trauma (approximately 90% of cases) or iatrogenic (<1%) [1-6]. Signs and symptoms of CJFs include dilated superficial veins, pulsatile neck swelling, and a palpable thrill, which are due to hemodynamic alterations resulting from the shunting of blood flow between arterial and venous circuits. Physiological adaptations to compensate for increased venous return and cardiac output may ultimately lead to irreversible high-output cardiac failure [7]. This case report details the presentation, diagnosis, and surgical intervention of a 66-year-old male patient presenting with an iatrogenic right CJF presumed to be due to previous central catheter placement versus guide wire trauma from repair of thoracic aortic dissection.

## Case presentation

A 66-year-old male patient presented to the clinic in March 2024 for evaluation of a right neck pulsatile mass. He reported experiencing a sensation of water rushing in his neck and "cracking sounds" when turning his head to the left for at least one month prior to presentation. The patient denied stroke-like symptoms, headaches, vision changes, neck swelling, or facial congestion. The patient's past medical history included a prior cerebrovascular accident without residual symptoms, hypertension, hyperlipidemia, peripheral artery disease, type 2 diabetes mellitus, tobacco abuse, and polysubstance abuse. Additionally, he had an extensive surgical history, including a thoracic endovascular aortic repair for a type B aortic aneurysm in November 2021 and a femoral-femoral bypass for severe limb claudication secondary to chronic extensive iliac occlusive disease in December 2021. Of note, the patient was admitted to an outside facility approximately three months prior to initial presentation, at which time he was admitted to the intensive care unit (ICU) requiring placement of a central line in the right internal jugular vein. Unfortunately, we were unable to obtain records from the patient's previous hospital stay to confirm the duration of central line utilization. 

**Figure 1:**
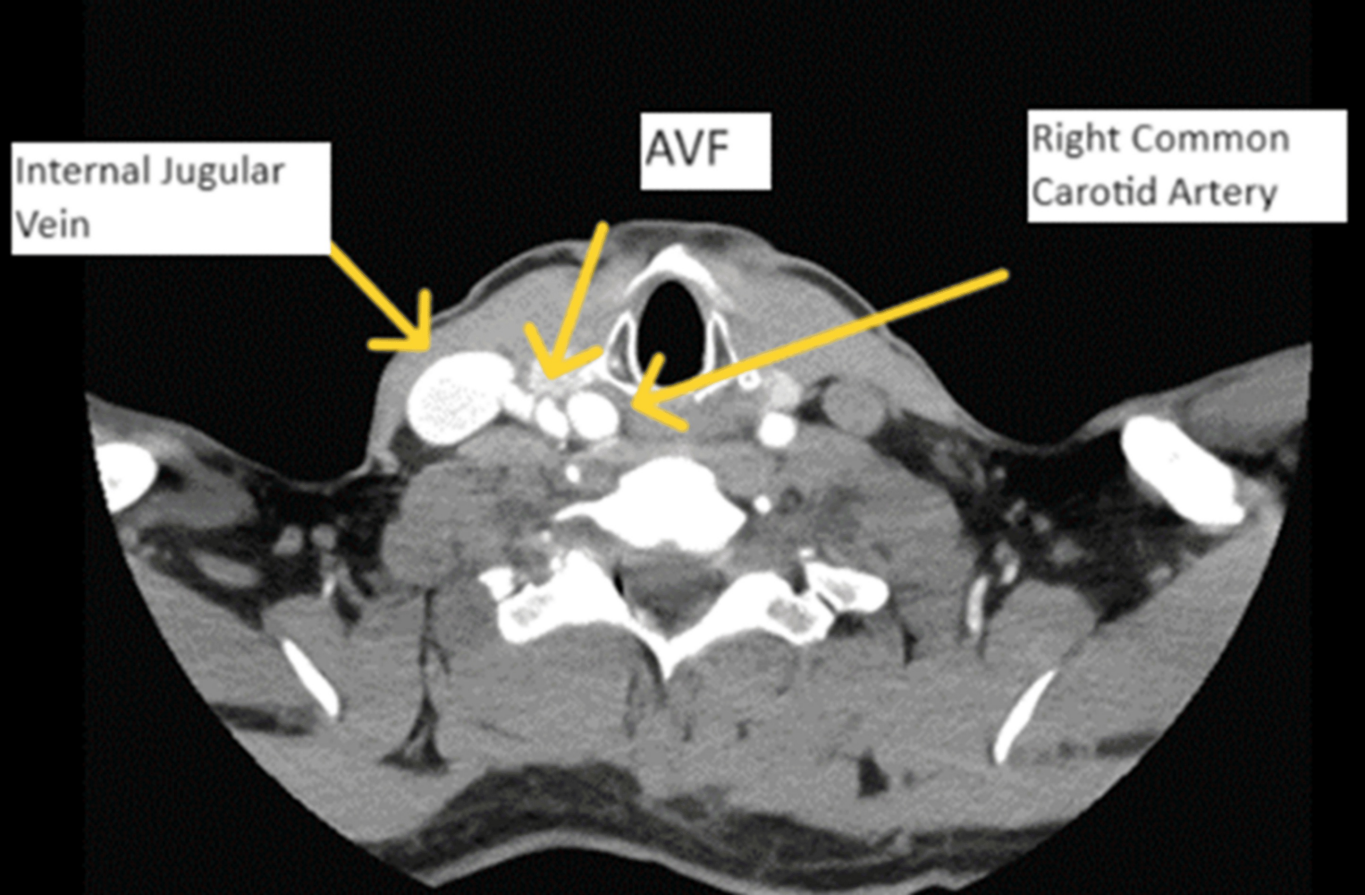
Computed tomography angiography head/neck with contrast depicting the carotid-jugular arteriovenous fistula AVF: arteriovenous fistula

**Figure 2:**
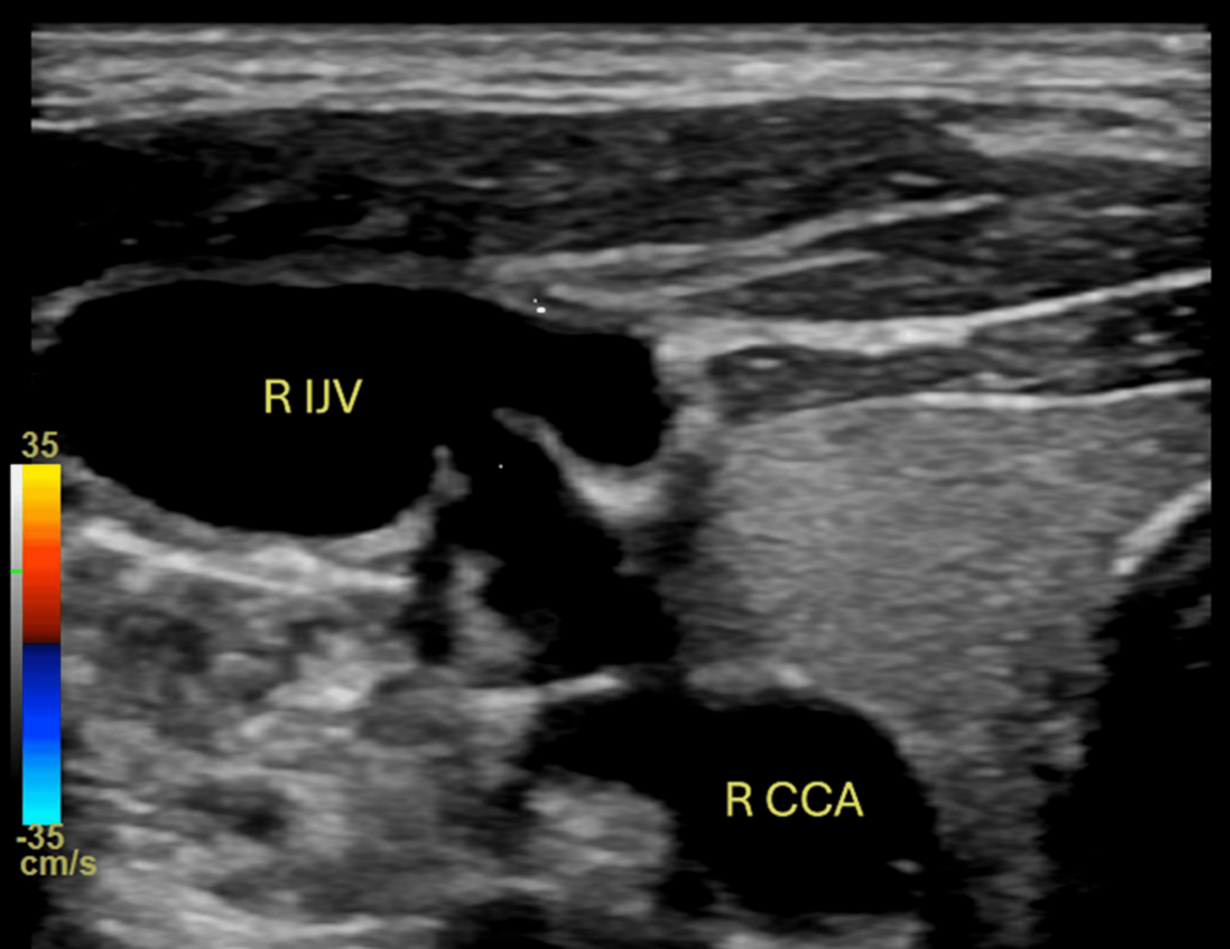
Carotid duplex ultrasound (grey scale) R IJV: right internal jugular vein; R CCA: right common carotid artery

**Figure 3:**
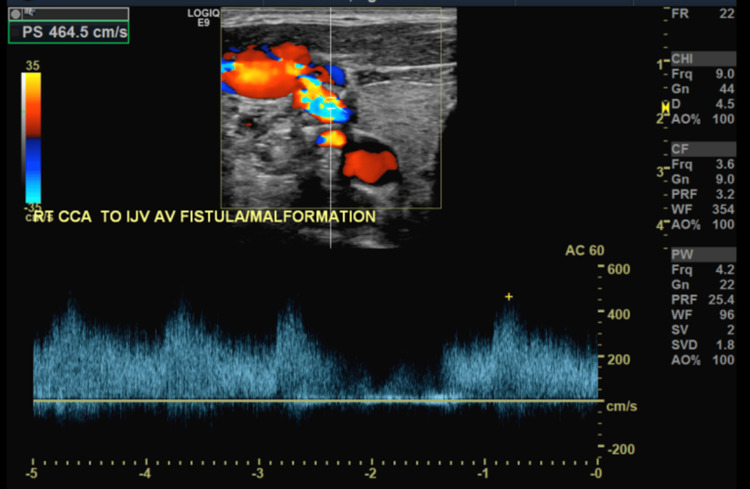
Carotid duplex ultrasound (color flow Doppler) R IJV: right internal jugular vein; R CCA: right common carotid artery; AV: arteriovenous

CT angiography (CTA) head/neck was obtained and demonstrated a 16 x 10 mm aneurysm versus pseudoaneurysm at the posterior lateral aspect of the right common carotid artery (Figure 1). Imaging results were discussed among the vascular surgery team, and it was concluded that rather than an aneurysm or pseudoaneurysm, this imaging finding was more likely representative of a CJF. Therefore, additional imaging was warranted. Carotid duplex ultrasound (Figures 2, 3) was performed and demonstrated an AV malformation within the right proximal common carotid artery to the internal jugular vein corresponding to the CTA findings.

Imaging findings were discussed with the patient, and he was offered surgical repair. After discussing the risks and benefits of operative intervention, informed consent was obtained from the patient. Given the patient’s significant past medical and surgical history, he went under extensive cardiac risk stratification and optimization at our facility and was then cleared for surgery.

Surgical intervention

Under general anesthesia, the patient was positioned supine on the operating room table with a shoulder roll between his shoulder blades to ensure adequate neck hyperextension. The patient’s neck was prepped and draped in the usual sterile fashion. A 5 cm incision was made anterior to the sternocleidomastoid (SCM) muscle. Dissection proceeded through Scarpa’s fascia and platysma, with the medial fascial attachment of the SCM divided and the muscle retracted laterally. The anterior cervical fascia was identified and dissected to expose the internal jugular vein and common carotid artery (CCA), which were controlled with ligatures.

Circumferential dissection of the CCA was performed proximal to the incision line, just above the clavicle. Careful dissection proceeded toward the fistula, clearing the connection cephalad and caudad. Control posterior to the fistula was not achievable initially. Therapeutic dosages of heparin were administered, and mean arterial pressure (MAP) was maintained above 80 mmHg.

**Figure 4:**
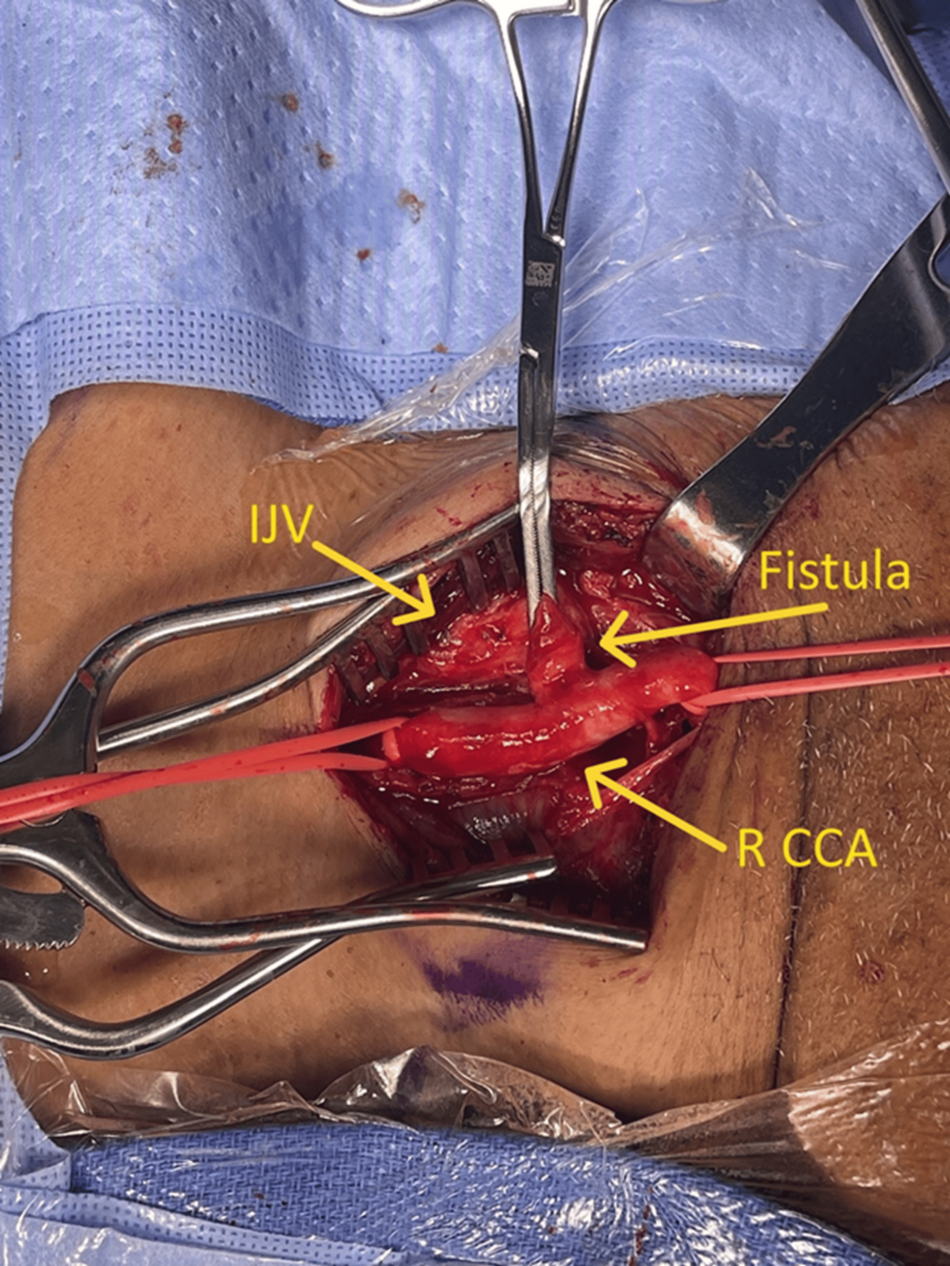
Intraoperative right carotid-jugular fistula R CCA: right common carotid artery; IJV:  internal jugular vein

**Figure 5:**
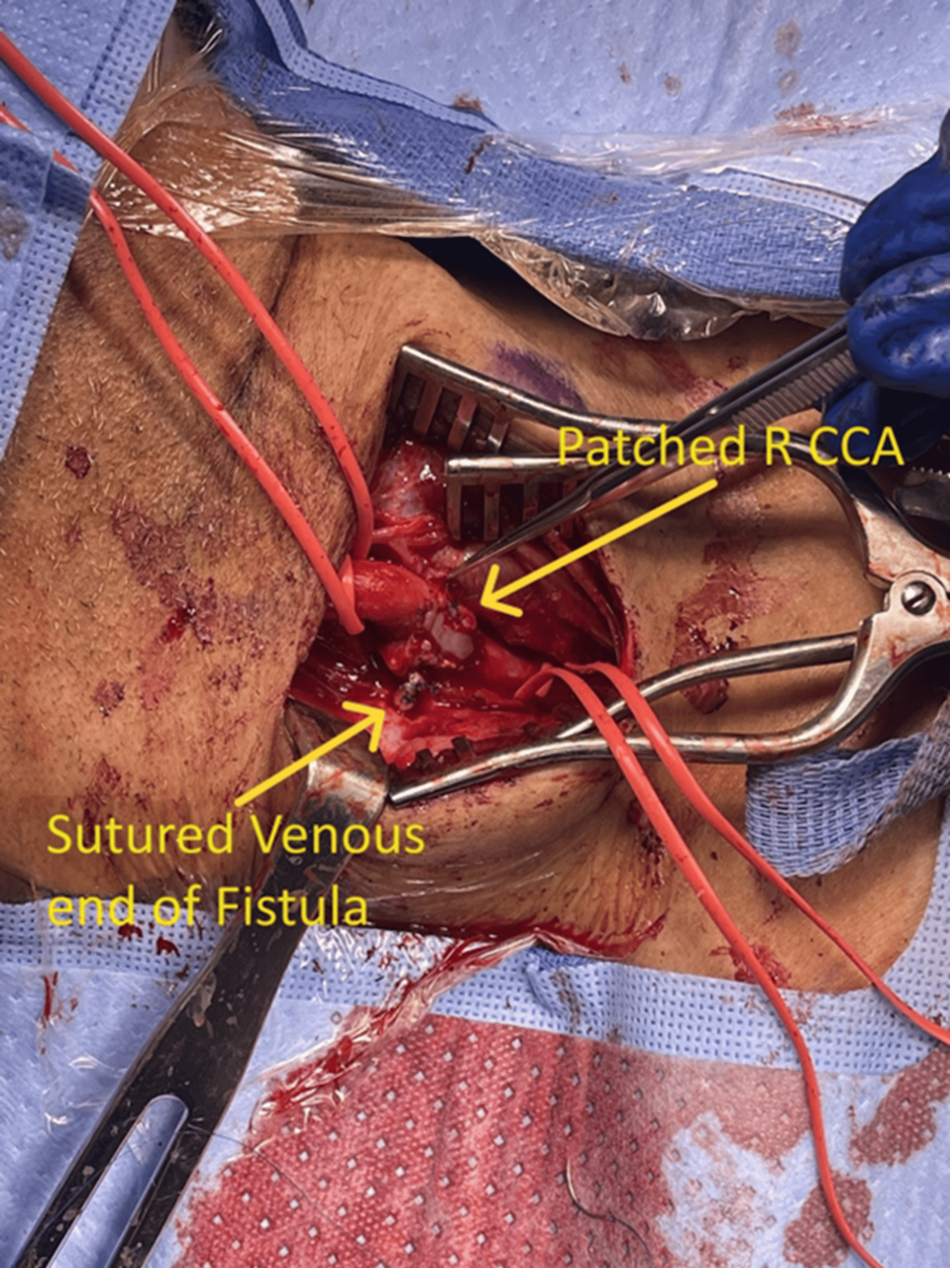
Intraoperative arteriotomy with bovine patch angioplasty R CCA: right common carotid artery

The fistula was clamped and divided between the clamp and the CCA (Figure 4). The disrupted intima was tacked down with sutures, and the arteriotomy was repaired using a bovine pericardial patch to prevent artery narrowing (Figure 5). The artery was allowed to bleed forward and backward with heparinized saline flush before closure to check for leaks. The venous end of the fistula was sutured closed.

**Figure 6:**
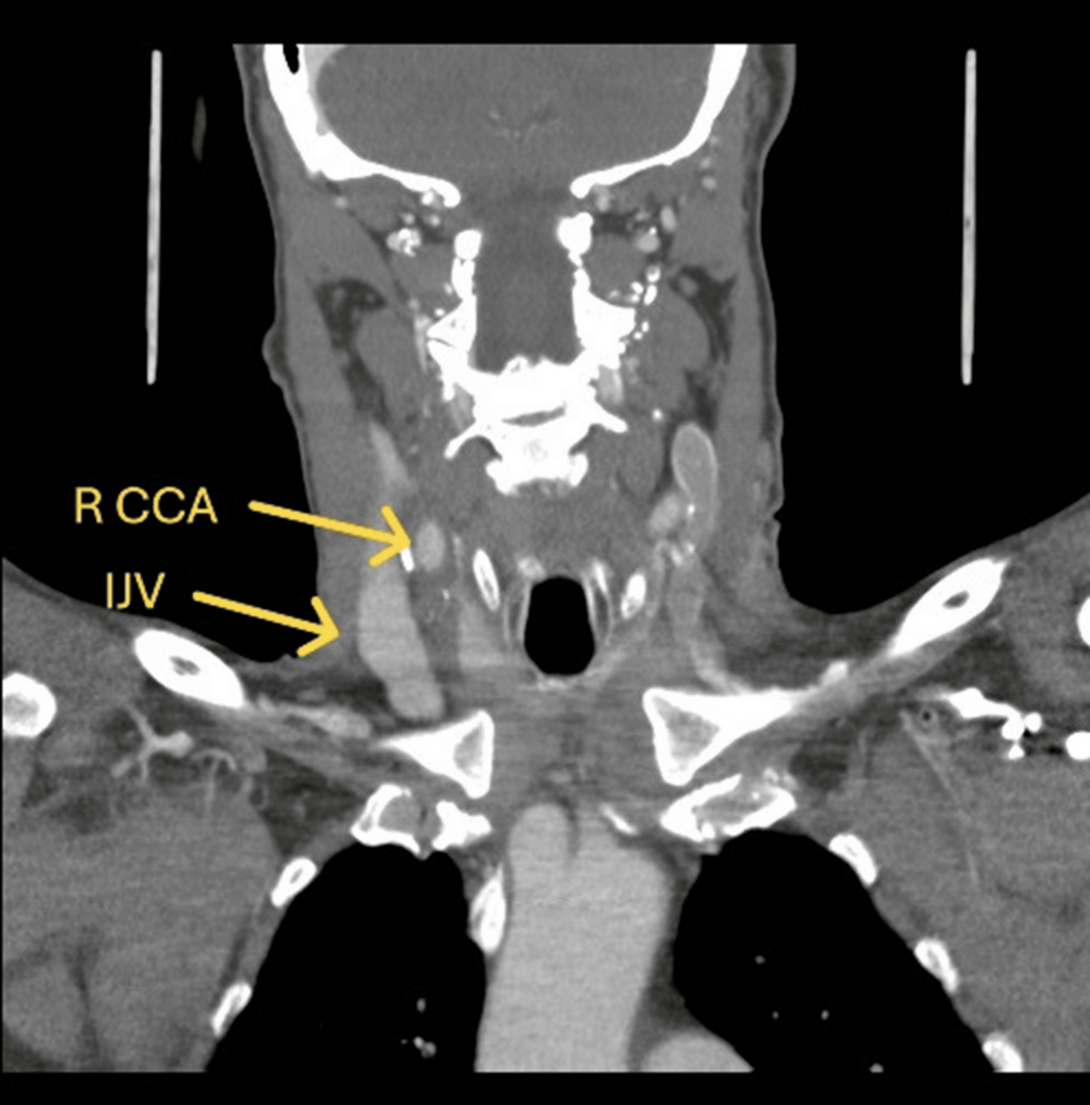
Postoperative computed tomography-angiography showing no persistent fistula R CCA: right common carotid artery; IJV: internal jugular vein

Hemostasis was achieved, and heparin was reversed with protamine. A continuous wave Doppler ensured proper flow. The SCM, platysma, and skin were closed in two layers. A Jackson-Pratt drain was stitched in place, and the incision was covered with a sterile dressing. The patient was extubated, neurologically assessed without focal deficits, and transferred to recovery in stable condition. He was then relocated to the surgical ICU overnight for close observation. The patient’s hospital course was otherwise uncomplicated, and no acute events were noted postoperatively. Given the absence of focal neurologic deficits, the patient's drain was removed, and he was medically cleared for discharge after meeting all discharge criteria and milestones. The patient was discharged on the second postoperative day. Patient was seen in follow since then and has healed the surgical site and remains neurologically intact. His follow-up neck CTA (Figure 6) shows intact carotid artery repair with no pseudoaneurysm.

## Discussion

Acquired CJFs are uncommon and account for approximately 4% to 7% of all traumatic AVFs [4,5]. CJFs develop as rare complications secondary to penetrating neck trauma or central line catheterization for hemodialysis, parenteral nutrition, and drug delivery [7]. When compared to AVFs located within extremities, CJFs are prone to complications such as intractable high-output cardiac failure, atrial fibrillation, and embolization [4-7]. Therefore, a high index of suspicion is required as fistulas are often missed during the acute phase of injury, and most patients are not treated for weeks or months after the initial injury.

Hemodynamic effects are important sequelae of AVFs, and the degree of arteriovenous shunting depends on the location, size, and distensibility of the fistula [5,7]. Large AVFs can reduce total peripheral vascular resistance, increasing cardiac output and potentially causing high-output cardiac failure in patients with poor cardiac reserves [8,9]. CT angiography remains the gold standard for AVF diagnosis, while magnetic resonance angiography and carotid Doppler ultrasound are also valuable [10]. Early intervention is crucial to prevent complications, as most AVFs enlarge over time [11]. 

Given that internal CJFs occur infrequently, appropriate procedural standards or definitive surgical techniques have not yet been established. Treatment options for AVFs include embolization, endovascular stenting, and open surgical repair, chosen based on the fistula's nature. Generally, embolization or endovascular interventions are suitable for complex fistulas with multiple draining vessels, while open surgery is preferred for simple, large, and superficial fistulas [5]. In our case, open surgical repair was preferred due to the tortuous course and location of the patient’s CJF. Although the fistula was initially difficult to isolate, we were successfully able to dissect the entirety of the CJF from surrounding anatomic structures without significant nerve injury or blood loss. Compared to endovascular repair, the open approach may increase risk for major blood loss and nerve injury as location and size can make fistula exposure difficult [12]. Therefore, given the complexity of open repair due to increased risk, the endovascular approach is more favorable. However, caution must be used during endovascular intervention due to the potential use of coils or plugs, which are associated with a high risk of pulmonary and cerebral embolization [4]. The lack of guidelines and literature makes the approach to AVF repair dependent on surgeon preference, expertise, and the circumstances of the specific AVF.

## Conclusions

AVFs are rare yet serious complications of central line catheterization, requiring high suspicion for early detection and intervention to prevent complications such as atrial fibrillation, embolization, infection, and high-output cardiac failure. This case report highlights the presentation, diagnosis, and successful surgical intervention in the treatment of carotid-internal jugular fistula.

## References

[1] Droll KP, Lossing AG (2004). Carotid-jugular arteriovenous fistula: case report of an iatrogenic complication following internal jugular vein catheterization. J Clin Anesth.

[2] Henry TC, Huei TJ, Yuzaidi M (2020). Unexpected complication of arteriovenous fistula of the left common carotid to internal jugular vein following central venous catheterization. Chin J Traumatol.

[3] Narroway HG, Bourke B, Tchen AS (2022). Acquired carotid-jugular fistula secondary to a pseudoaneurysm following carotid endarterectomy. Vasc Endovascular Surg.

[4] Puca AE, Pignatelli F (2015). An adult case of idiopathic internal carotid-internal jugular vein arteriovenous fistula. Ann Vasc Surg.

[5] Steiger K, Ritchie C, Pollak PM, Sandhu SJ, Miller D, Erben Y (2022). Iatrogenic left common carotid artery to right internal jugular vein arteriovenous fistula closure. J Vasc Surg Cases Innov Tech.

[6] Ezemba N, Ekpe EE, Ezike HA, Anyanwu CH (2006). Traumatic common carotid-jugular fistula: report of 2 cases. Tex Heart Inst J.

[7] Caldarelli C, Biricotti M, Materazzi G, Spinelli C, Spisni R (2013). Acquired carotid-jugular fistula: its changing history and management. ISRN Surg.

[8] Erdöl C, Baykan M, Gökçe M (2002). Congestive heart failure associated with chronic venous insufficiency and leg ulcers secondary to an arteriovenous fistula caused by a shotgun wound 15 years ago. Vasa.

[9] Robbs JV, Carrim AA, Kadwa AM, Mars M (1994). Traumatic arteriovenous fistula: experience with 202 patients. Br J Surg.

[10] Patel R, Nicholson AA (2012). Arteriovenous fistulas: etiology and treatment. Endovasc Today.

[11] Talwar S, Bhan A, Sharma R, Choudhary SK, Venugopal P (2000). Carotid artery-to-jugular vein fistula. Asian Cardiovasc Thorac Ann.

[12] Wang M, Fan W, Mungur R, Gu J, Wan S (2018). Endovascular treatment of congenital internal carotid-jugular fistula. Front Neurol.

